# Gender matters: factors important for quality of life in midlife after stroke

**DOI:** 10.3389/fneur.2025.1590900

**Published:** 2025-09-04

**Authors:** Marie Matérne, Gustav Jarl, Grahame Simpson, Peter Appelros, Ingrid Thermaenius, Mialinn Arvidsson Lindvall

**Affiliations:** ^1^School of Behavioural, Social and Legal Sciences, Örebro University, Örebro, Sweden; ^2^University Health Care Research Center, Faculty of Medicine and Health, Örebro University, Örebro, Sweden; ^3^Department of Prosthetics and Orthotics, Faculty of Medicine and Health, Örebro University, Örebro, Sweden; ^4^School of Health Sciences, Faculty of Medicine and Health, The University of Sydney, Camperdown, NSW, Australia; ^5^Department of Health Care Sciences, Marie Cederschiöld University, Stockholm, Sweden; ^6^School of Health, Care and Social Welfare, Mälardalen University, Västerås, Sweden

**Keywords:** gender, mid-life, lifespan development, quality of life, stroke

## Abstract

**Background:**

Coping with disabilities after stroke in midlife can be challenging, with potential gender differences that may have implications for quality of life (QoL) and support. This study aimed to explore QoL and resilience among midlife stroke survivors from a gender perspective.

**Methods:**

Quantitative questionnaire data related to demographics, function, service, resilience and QoL were gathered from a stroke register including 51 individuals (of whom 29 were men) aged 40–64 years. Results of gender were compared using two-sided *t*-tests and chi-square tests. Additionally, eight semi-structured telephone interviews were conducted, with equal representation of men and women. Qualitative content analysis was used to explore deeper and capture nuanced insights.

**Results:**

The quantitative analysis revealed no statistically significant gender differences. However, the qualitative data revealed three central themes: (1) “A Forced Lifestyle Change,” (2) “Lack of Understanding and Support,” and (3) “Importance of Independence and Coping Strategies.” Men talked about feelings of being restricted in their post-stroke lives and expressed a greater need for support from healthcare providers, family, and friends. In contrast, women described having more well-developed coping strategies and reported a higher perceived QoL.

**Conclusion:**

Qualitative findings suggest men may face greater challenges in adapting to post stroke life. The result suggests that men struggle with accepting limitations that prevent them from participating in social contexts and require more support from healthcare services. These difficulties, potentially due to less effective coping mechanisms, may result in a lower QoL. Gender-sensitive interventions addressing these needs could improve QoL and adaptation.

## Introduction

The consequences of a stroke, which often last a lifetime, are multifaceted and bring different challenges in life (1). Gender differences have been reported regarding stroke types and severity, mortality rates, degree of disability and quality of life (QoL) (2–4). Overall, women tend to have more significant disabilities than men (3, 4). These differences can then flow through to gender-based distinctions in rehabilitation outcomes with women having a somewhat less favorable prognosis (5, 6). The reason for these disparities can be sought in the above stroke characteristics, but also due to variations regarding depression and quality of care (6, 7) amongst other factors.

The impact of stroke can have wide-ranging effects on an individual’s life, and coping with its challenges often requires various strategies that significantly influence psychological health and wellbeing (8). There are noticeable gender differences in health-related quality of life; several studies have found that women tend to have lower mean score than men following a stroke (9–11). The majority of research has focused on the stroke population as a whole, meaning that the age range is strongly skewed towards people over the age of 65 years (3, 4), thereby obscuring the potential impact on particular age-related sub-groups. For example, there is limited research about the consequences of stroke in midlife (i.e., between 40 and 65 years), an important stage within adult life often overlooked by researchers (12).

Midlife is a pivotal part of the lifespan, balancing both growth and decline, and linking earlier and later parts of life (12). People in midlife might be juggling multiple demands and stressors (13), whilst dealing with newly arising physical and cognitive challenges. It can also be a time of personal, family, financial, and occupational success (14). Furthermore, better physical health in midlife can lay the foundation for good health in old age (15). Within this context, stroke might be seen as a non-normative event within the midlife age span, with implications for how its impact is perceived and experienced.

Previous studies focusing on gender differences have predominately taken a quantitative approach and focused on an older, or a mixed age sample (2–4, 6, 7, 10). The current study specifically examined the impact of stroke on participants in midlife from two perspectives. First, demographic, functional, service-related, resilience, and QoL data were collected and analyzed to provide a broad overview and identify gender-related patterns and trends. To deepen this analysis, experiences of living with stroke in midlife were explored through qualitative interviews among a subset of the sample.

## Materials and methods

This study is based on the Kumla stroke register, which represents all stroke survivors in a medium-sized Swedish municipality. Quantitative data, focusing on QoL and participation, were retrieved from a stroke assessment battery (16, 17). The qualitative interviews were based on a sample of eight participants.

### Participants and setting

The study is part of a lager cohort of stroke survivors living in Kumla, a medium-sized Swedish municipality. The data are based on the Swedish stroke register (Riksstroke), and of the medical record system of Region Örebro (18). By December 31, 2019, a total of 330 persons were identified living as stroke survivors in the municipality including all age groups. Their diagnosis was verified from data in patient records by one of the co-authors (PA), a neurologist (16). An information letter describing the study, a consent form, and the study questionnaires were sent by post to all these persons. In this study only individuals aged 40–65 years were included in the quantitative analysis (*n* = 51, 15% of the total population), as 65 marks the retirement age in Sweden, and the aim was to focus on participants in midlife.

### Quantitative data and analysis

#### Measures

*The SF-36* is a self-report measure of QoL in eight domains, each scored from 0 to 100 with a higher score reflecting higher QoL. In previous validation studies, the Swedish version of SF-36 has demonstrated strong internal consistency (*α* > 0.80) for the majority of socio-demographic subgroups (19).

*The CD-RISC 25* is a 25-item self-report measure of resilience, producing a sum score from 0 to 100 where a higher score indicates a higher level of resilience. The original CD-RISC 25 has demonstrated strong internal consistency (*α* = 0.93) and test–retest reliability (20), and the Swedish translation of CD-RISC has also demonstrated sound psychometric properties (21).

*The Riksstroke 1-year follow-up questionnaire* is a self-report measure constructed by the Swedish Stroke Register (Riksstroke), and is used for long-term follow-up of stroke patients. It has demonstrated content and face validity (22). The measure includes four domains: (i) Accommodation and activities in daily living, (ii) Support or help from healthcare or municipality services, (iii) Rehabilitation after discharge from hospital, and (iv) state of health and care interventions. This study included demographic, functional, and service-related items from the Riksstroke questionnaire.

#### Analysis

Data was entered into IBM SPSS Statistics version 29 for the quantitative analyses. Missing values on CD-RISC items were imputed for *n* = 3 cases (<5%) using the mean value of the participant’s answers on the other CD-RISC items. SF-36 domain scores were estimated for each participant with less than 50% missing values on the domain.

Descriptive statistics were generated for demographic and stroke related variables, as well as for all three measures. SF-36 domain scores and CD-RISC scores were compared using a two-sided *t*-test with 95% confidence limits. Response categories on the Riksstroke questionnaire were dichotomized due to the small sample size. Given the modest sample sizes, Fischer’s exact test was used for all items as the expected cell count was <5 for one or more cells. *p*-values <0.05 were considered to be statistically significant.

### Qualitative data and analysis

The informants were selected from interviews made within the Kumla stroke study (23). Eight individuals (four women and four men) aged between 45 and 60 years were interviewed, all of them had been unable to return to full gainful employment. When selecting the eight interviews, we aimed for heterogeneity in gender and age. These eight interviews were selected from another interview sample (23), to be completely re-analyzed from a gender perspective, a perspective that was not part of the original study. The residual stroke symptoms were based on the informants’ responses in the interviews.

All eight interviews were conducted by telephone, due to the COVID pandemic, seven by IT and one by MM, both registered healthcare counselors with extensive experience. A semi-structured interview guide covering four areas was used: (1) Background (age, gender, employment, diagnoses), (2) QoL, (3) Participation and treatment, and (4) Resilience (Appendix 1). The guide was piloted on one stroke survivor (not part of the study) to verify comprehensibility. No changes were made after the pilot test. Each interview lasted for about 30–45 min. The interviews were performed between May 15 and June 11, 2020. In one interview, the informant had aphasia, and his wife provided support during the interview. All interviews were recorded and transcribed verbatim.

#### Analysis

For the analysis, we employed qualitative content analysis (24). We adopted an inductive approach with the objective of exploring perspectives of QoL and participation. Initially, the transcribed text was read several times by IT, MM, and MLA. The data were structured in tabular form in a word document. The analysis continued with identifying meaningful units in the transcribed interview material, without analyzing or searching for any gender perspective. IT and MM constructed meaning units for the first two interviews, and IT analyzed the remaining six. In the next step, the meaning units were condensed by summarizing the text (24). The condensed meaning units were coded and marked with each participant’s gender. The codes were organized into categories at a latent level with interpretation by IT. The authors IT, MM, and MLA were engaged in the discussion in order to validate the results.

The last step was to assemble categories into themes. IT, MM, and MLA first worked independently. Consensus was then achieved in identifying the construction of the themes. The structure of the data and the naming of categories and themes were discussed within the whole research group until consensus was reached.

## Results

### Results from quantitative analysis

The total sample (*n* = 51) had a mean age of 55.2 (SD 6.4) years. The men (*n* = 29) were on average 55.6 (6.6) years and the women (*n* = 22) were 54.8 (6.3) years (*p* = 0.66). Chi-square analysis found no significant difference between men and women in terms of stroke type (22 men and 13 women had ischemic stroke; seven men and nine women had hemorrhagic stroke, intracerebral or subarachnoid hemorrhage) (*p* = 0.24). *T*-test found no significant difference in the average time since the first stroke for men [4.3 (SD 5.6) years] and women [4.7 (4.9) years] (*p* = 0.77).

### Quality of life, resilience, and Riksstroke items

Twenty-six participants answered the Short-Form 36 (SF-36), 22 answered the Connor-Davidson Resilience Scale (CD-RISC 25), and 28 answered the Riksstroke 1-year follow-up questionnaire (16, 17). The high dropout rate can be attributed to the fact that, although consent was collected in advance, not everyone was willing to proceed with the tests. However, many still chose to complete the questionnaire. Additionally, the process of completing both the questionnaire and the test was time-consuming, which may have deterred some participants.

Results from the between-groups analysis of the SF-36 domains (total *n* = 26, men = 16, women = 10) are displayed in Table 1. Gender differences in raw scores ranged from women having 14.1 points higher score on the Role-physical domain to men having 13.4 points higher score on the Physical functioning domain (Table 2). However, no differences in domain scores were statistically significant (*p* = 0.39–0.99). Resilience scores, as measured with CD-RISC (*n* = 22), found a large disparity between men and women (see Table 1), and there was a trend for the difference of 16.5 points to be statistically significant (*p* = 0.074).

**Table 1:** Comparisons of quality of life and resilience between men and women.

Instruments	All, mean (SD)	Men, mean (SD)	Women, mean (SD)	Mean difference, men–women (95% CI)	*p*-value
SF-36 domains, score 0–100^a^					
Physical functioning^b^	66.7 (33.6)	71.9 (26.8)	58.5 (42.7)	13.4 (−19.1; 45.8)	0.39
Role-physical	66.0 (41.4)	60.9 (43.8)	75.0 (37.5)	−14.1 (−50.0; 21.9)	0.43
Bodily pain	62.2 (30.2)	62.3 (31.0)	62.1 (30.4)	0.2 (−25.5; 25.8)	0.99
General health	62.8 (24.9)	65.2 (22.2)	59.0 (29.6)	6.2 (−14.8; 27.2)	0.55
Vitality	57.1 (25.9)	60.0 (25.1)	52.5 (27.8)	7.5 (−14.3; 29.3)	0.48
Social functioning	74.0 (29.3)	75.8 (25.6)	70.8 (36.4)	4.9 (−20.8; 30.7)	0.69
Role-emotional	63.9 (43.9)	64.6 (46.3)	62.5 (41.5)	2.1 (−38.2; 42.4)	0.92
Mental health	75.4 (22.0)	74.3 (23.9)	77.2 (19.6)	−3.0 (−21.6; 15.7)	0.75
CD-RISC, score 0–100^c^	63.2 (21.4)	69.9 (17.3)	53.4 (24.0)	16.5 (−1.8; 34.8)	0.074

a All *n* = 26 (men *n* = 16; women *n* = 10), SF-36, 36-Item Short Form Survey; SD, standard deviation; CI, confidence interval, CD-RISC, Connor Davidson resilience scale.

b Only for one comparison, Leven’s test for equality of variances had a *p*-value <0.05 (*p* = 0.007). Thus, for this comparison, *t*-test with equal variances not assumed was used. *T*-test with equal variances assumed was used for all other comparisons.

c All *n* = 22 (men *n* = 13, women *n* = 9).

**Table 2:** Comparisons of Riksstroke questionnaire items between men (*n* = 17) and women (*n* = 11).

Items from Riksstroke questionnaire	Response categories	All, *n* (%)	Men, *n* (%)	Women, *n* (%)	*p*-value^a^
2. Do you live alone?	Yes	8 (29)	5 (29)	3 (27)	1.00
	No	20 (71)	12 (71)	8 (73)	
3. Do you still have problems after your stroke?	Yes	21 (81)	11 (69)	10 (100)	0.12
	No	5 (19)	5 (31)	0 (0)	
4. Have you been able to return to your pre-stroke life and activities?	Yes	11 (39)	6 (35)	5 (45)	0.70
	Yes, but not as before/no	17 (61)	11 (65)	6 (55)	
10. Do you think that your needs for support or help from healthcare or the municipality are met after your stroke?^b^	Did not need or did not want support or help/yes, completely fulfilled	16 (59)	10 (59)	6 (60)	1.00
	Yes, partly met/no, not met	11 (41)	7 (41)	4 (40)	
18. Do you know where to turn if you need support or help after your stroke?	Yes	18 (64)	9 (53)	9 (82)	0.23
	No/do not know	10 (36)	8 (47)	2 (18)	
20. Satisfaction with rehab or training after hospital discharge (because of your stroke)^b^	Satisfied/very satisfied/did not need rehab or training after hospital stay	16 (64)	10 (59)	6 (75)	0.66
	Dissatisfied/very dissatisfied/needed, but did not get rehab or training after hospital stay	9 (36)	7 (41)	2 (25)	
22. Dependent on support or help from family members/relatives^b^^,^^d^	Yes, completely dependent/yes, partly dependent	10 (36)	6 (35)	4 (36)	1.00
	Not at all	18 (64)	11 (65)	7 (64)	
23. Returned to gainful employment	Yes, to the same extent as pre-stroke/yes, but to a lesser extent than pre-stroke	17 (71)	10 (67)	7 (78)	0.67
	No, but planning to return to gainful employment/no, cannot return to gainful employment	7 (29)	5 (33)	2 (22)	
24. Have you received work-oriented rehabilitation after the stroke?^c^	Has returned to gainful employment without work-oriented rehabilitation/yes, have received work-oriented rehabilitation to a large extent	15 (65)	10 (67)	5 (63)	1.00
	Yes, have received work-oriented rehabilitation but not enough/no, not at all, but needs work-oriented rehabilitation	8 (35)	5 (33)	3 (38)	

a Fischer’s exact test.

b Do not know” answers were treated as missing.

c “No, not applicable. Have a pension or were not gainfully employed before suffering a stroke” answers were treated as missing.

d “Has no relatives or lacks contact with relatives” answers were treated as missing.

Finally, analyses of the informant responses (*n* = 28) on the Riksstroke items employing Fisher’s Exact test did not find any significant gender-based differences (*p* = 0.12–1.0) (see Table 2). Five of 17 men (29%) and three of 11 women (27%) lived alone (chi-sq ns, *p* = 1.0). Furthermore, there were no differences in the proportion of men versus women able to return to gainful employment post-stroke (10 of 15 men, 67%; 7 of 9 women, 78%; chi-sq ns, *p* = 0.67).

### Results from the interviews

The profile of interview participants is displayed in Table 3.

**Table 3:** Profile of interviewees.

Name	Age	Sex	Marital status	Years since stroke	Self-reported residual symptoms	Occupation
Anna	60	F	Single	8	Spasticity, can walk with a stick, wheelchair user	SC
Gerd	45	F	Partner	2	Right side paralysis, fatigue	SC, Part time administrator
Erika	55	F	Single	2	Numbness, right-side paralysis	SC, Part time human resource manager
Caroline	58	F	Partner	6	Fatigue, memory loss, aphasia	SC, Part-time assistant at school
Henric	57	M	Partner	19	Balance, dizziness, fatigue, memory loss, mood swings	SC, Part time finance assistant (with a salary compensation)
Fredrik^a^	57	M	Partner	3	Right-side paralysis, memory loss, aphasia	SC
Daniel	45	M	Single	1	Double vision	SC, Work training
Björn	60	M	Partner	11	Left-side paralysis, spasticity	SC

SC, sickness compensation. The names of the informants are pseudonyms to protect their anonymity.

a Wife participated in the interview.

Content analysis elicited three overarching themes from the participants’ experiences, namely: (1) A forced lifestyle change, (2) Lack of understanding and support, and (3) Importance of independence and coping strategies. Each theme consisted of three categories (Table 4). Different gender perspectives were identified in some but not all the categories, marked in the table and in the text with *. All categories are discussed below with citations from the participants to illustrate the gender perspectives.

**Table 4:** Themes and categories.

Themes	A forced lifestyle change	Lack of understanding and support	Importance of independence and coping strategies
Categories	Employment and leisure activities are affected	Relationships have changed*	Quality of life has changed*
	Participation has to be adapted	Support from relatives is valuable	Continued independence is important
	Accessibility is reduced in everyday life	Lack of support from healthcare*	Coping strategies are central*

*Categories with gender differences identified.

### A forced lifestyle change

In the first theme, three categories were identified, where women and men described their forced lifestyle changes differently.

#### Employment and leisure activities are affected

Adjustments in the performance of activities were common in both women and men, although their leisure interests were the same as before the stroke. Some of the participants had to reduce their activities due to fatigue, while others were dependent on support to be able to pursue their interests. Having practical help from a partner or personal assistant was described as facilitating by both women and men. This additional support helped them to continue with their existing hobbies. Fredrik continued with his photography hobby but was using equipment that was easier to handle with his hemiplegia:

*‘You still have the interest in photography and when we are out you take photos with a slightly smaller camera and with the camera in your mobile’ (Fredrik’s wife).*

Men placed a greater emphasis on feeling limited in everyday life compared to the women. Men observed that they had to either slow down or change their activities, whereas the women talked about an adaptation to their new situation rather than a feeling of limitation. The women were more sensitive to stress, and preferred activities at home to reduce stress in their lives.

I like to potter about at home, with flowers and things like that. Yes, I danced a lot before, at an elite level. And then, of course, there’s my kids that I care about a lot that I spend a lot of time with. In any case my son, my daughter lives down in xx of course, but my son lives up here. And they mean a lot to me (Caroline, 58).

#### Participation has to be adapted

Experiences of negatively affected possibilities of participation in everyday life was commonly in both women and men. They described experiences of having low energy from dizziness, fatigue, and other physical limitations, which restricted them to participate. However, they tried to be involved in everyday life as much as possible. Some, both women and men also expressed pride in having come as far as they had, despite the challenges post stroke.

#### Accessibility is reduced in everyday life

Accessibility was expressed as physical, informational, and communication, however no specific differences between the genders were identified. Two participants (one woman, one man) were dependent on assistive devices outdoors. Fredrik experienced that his access to participating in society was limited. It was especially difficult when visiting friends whose accommodation was not adapted for wheelchairs. When he was going to a new environment that he had no information about in advance, he felt insecure. Fredrik’s wife saw this as a major obstacle for him, and it affected his participation:

*“Wherever we go, you have to call in advance and check if it is adapted for people with disabilities. If there is a toilet and if you can get in with a wheelchair and … access is often limited’ (Fredrik’s wife).*

### Lack of understanding and support

The second theme was about understanding and support, both from close relatives and from the healthcare service. In this theme, three categories were identified. The men described more changes in social relationships and men also wanted more support and follow-ups from the healthcare system.

#### Relationships have changed*

Men shared that they experienced more complexity and challenges in their relationships and that their circle of friends had decreased. One man observed that he and his wife were no longer invited to acquaintances’ houses, whereas close friends did not treat them differently. Some of the men stated that it was difficult to make new friends. They thought that this was due to their stroke making people a little cautious because they did not know how to handle the situation. The women did not express the same complexity and challenges and stated:

*‘Yes, but there is no difference so, there is not. It makes no difference, because my friends and acquaintances know about it, or know what I’ve been through (Caroline, 58).’*

#### Support from relatives is valuable

Support from close relatives was highlighted by all. Erika talked about that it was essential that her family understood her situation and got information about her stroke:

*‘Yes, but it’s probably that you have had understanding (from your loved ones), I suppose it’s important (Erika, 56).’*

Commonly expressions of feeling loneliness and of not getting the support they needed were expressed by both genders, but men had more feelings of loneliness and lack of support than the women did. One man who lived alone and only had a few relatives thought he had a demanding situation, having to manage most daily tasks and activities unaided.

Both women and men thought that having children gave them strength to continue fighting and was a driving force in their fight to return to normal life.

That you, if you have a little daughter who is dependent on you, that is more than anything that has helped me. Had I been completely alone, I don’t know, if I’d been single, no /.../ it has helped me a lot mentally and /.../ I kind of have to get out of this (Daniel, 45).

#### Lack of support from healthcare*

Some men expressed feelings of receiving insufficient support from the healthcare service and they desired more comprehensive follow-up care. They felt abandoned after being discharged from the hospital, especially as they navigated significant life changes post stroke. The importance of psychosocial support was highlighted as central and important in post-stroke recovery. Björn emphasized the need for increased support from the healthcare service; he wanted them to contact him:

‘*No, so I would like more support from the hospital. To not just be discharged and then that’s it. It’s bad follow-up, I would say (Björn, 60).’*

Björn also described that he had to initiate contact himself when he sought support from the healthcare counselor after separating from his wife following the stroke. He believed that if healthcare had provided regular follow-ups, he could have reached out and discussed different difficulties earlier and perhaps felt less distressed.

### Importance of independence and coping strategies

In the final theme, experiences of how to cope with new situations in life after stroke was illustrated in three categories. In this theme, the men frequently expressed that their QoL had deteriorated, and that they faced challenges in developing coping strategies to adjust to their post-stroke life.

#### Quality of life has changed*

Impaired QoL was experienced by some men. Limitations such as fatigue, noise sensitivity, worse finances due to work limitations and not managing life as before were challenges several of the men talked about. They also said that the initial phase after stroke was mostly about surviving. One of the informants, Daniel, described how his QoL gradually improved as his physical and psychological recovery progressed.

Right now it [QoL] is probably unchanged, I have to say. Because it’s just as usual with my daughter. But there is still a difference this thing with fatigue and no job and. It is worse quality of life now, of course. I’m weakened, but it’s still no disaster, I’d say. But it’s worse, of course, much worse. So, but as good as it can get, you have to say (Daniel, 45).

Several of the women commented that their life situation had changed. However, they believed that not everything had become worse. They described that they coped with the situation and prioritized wellbeing.

#### Continued independence is important

Both women and men emphasized that being independent was central; they considered it important to be able to participate in family life, work, and leisure.

It is, of course, being able to do everything you could before, even now, and I do. I exercise a lot, training is important that I can work. And, and do things yourself, not having to have anyone’s help (Erika, 56).

Both women and men also said that wellbeing, having good health, being able to travel and to exercise, and continuing to be involved in activities were important for them to feel satisfied with life.

#### Coping strategies are central*

Several of the men highlighted the importance for them of physical and mental strength, and having a fighting spirit in their recovery. One man, Björn, had a background in sports and stressed that this was helpful as a coping strategy. He used his fighting spirit to never give up, keeping his focus forward and having clear goals, which were central to his recovery:

‘*Yes, I’m too stubborn for that. I have never done that [given up], then I would not have been where I am today (Björn, 60).’*

The women highlighted persistence, goal orientation and inner strength as important driving forces. One of them, Caroline, described how feelings of anger made her continue her recovery, but at the same time made her feel like giving up. Caroline reported that her social network thought that she was too hard on herself:

*‘My friends and acquaintances usually say that you must not use so much force on yourself, but I am so determined that I will return (Caroline, 58).’*

## Discussion

The study involved a small sample size, in both the quantitative and qualitative data, which limits the ability to draw broad conclusions from the results. Nonetheless, the study explored the impact of stroke on adults’ quality of life and resilience in midlife, aiming to broaden the gender perspective on how a stroke affected this age group. While the quantitative data found similarities between the genders in reporting the impact of stroke on their lives, the qualitative analysis uncovered differing narratives in the participants’ experiences. The four men in the qualitative interviews discussed challenges related to social changes, support from healthcare, and QoL. There were also examples of differing coping strategies between men and women. The men talked about struggles with the loss of social roles and support, whereas the four women recounted experienced social support and healthcare resources.

The male participants in the interviews expressed feelings of exclusion from social contexts, with some attributing this to their disabilities. This perceived exclusion might be linked to traditional gender roles, where men are often expected to embody strength and independence, fulfilling roles of breadwinners and family leaders (25). Experiencing a stroke could increase men’s dependence on others, potentially contributing to a decline in self-esteem and challenging their sense of identity (26, 27). The female participants did not report significant changes in their social treatment or relationships post-stroke. They did express that they still could find new contexts for participation. This difference between our male and female participants’ experiences might be interpreted in the context of longstanding societal expectations that position men as the primary providers and heads of their families (4). These traditional roles emphasis men’s central place in family and society, which could intensify feelings of inadequacy and loss of identity when such roles are disrupted. Meanwhile, some of the women expressed feelings of stress due to a burden, with responsibilities related to children and work. This phenomenon was less prominent among the men, which may reflect differing societal pressures and expectations on men and women (28).

The male participants talked about the value of returning to work and remaining active. They felt these factors significantly impacted their QoL, aligning with other studies that found individuals with acquired brain injury who were unable to return to work reported lower QoL (29–32). The men in our study also reported feeling more restricted in their work roles and needing to either reduce their working hours or stop working entirely. This suggests that employment may hold greater significance for men in maintaining their QoL than it does for women, consistent with other research which has found that return to work is easier for men and was associated with increases in QoL (31).

Our findings can also be interpreted through an intersectional lens. Intersectionality provides insight into how multiple social categories—such as disability and gender—intersect to create complex experiences of exclusion or discrimination. Some of the men in our study talked about a sense of isolation, feeling that their disability and restricted work participation led to exclusion and unequal treatment compared to others in society. This compounded effect of both disability and gender expectations may intensify perceived restrictions highlighting how societal roles and identity factors can intersect to shape individuals’ experiences of recovery and reintegration (33).

In the study, men talked about a greater need for support from healthcare services than women. A study on self-reported primary care visits found gender differences, with women reporting a higher frequency of seeking care for both physical and mental concerns (34). This difference may suggest that women are more attuned to recognizing and addressing health needs, while men may be less likely to seek care. One potential factor underlying men’s lower healthcare seeking behavior is the increased incidence of post-stroke depression among men, which may reduce their motivation or drive to pursue medical support (9, 35). This lack of drive could be exacerbated by societal expectations or internalized beliefs about masculinity, where men may feel discouraged from expressing vulnerability or seeking help for emotional or psychological issues (36).

Coping strategies and personal resilience were identified as important aspects of the recovery process by all participants. However, these strategies were more frequently discussed by the women, who provided examples of a broader range of coping techniques in the qualitative data. This finding aligns with previous research on other health conditions, which suggests that women tend to employ more diverse coping strategies (37). Coping strategies, such as developing a strong sense of coherence, can be particularly beneficial in helping individuals manage stress and adapt to changes after a life-altering event like a stroke (38). Sense of coherence, a concept that emphasizes comprehensibility, manageability, and meaningfulness, has been shown to enhance the ability to face and overcome stressors (39). Women’s greater reliance on a variety of coping mechanisms, potentially including emotional expression, social support, and active problem-solving, may enhance their resilience and positively impact their QoL. However, further research is needed to, particularly in the context of stroke in midlife, to draw any conclusions.

Future research should further explore gender differences in stroke recovery in midlife, an area with limited existing studies, particularly using mixed-method approaches. Larger studies are needed to validate these findings. Additionally, examining psychosocial support and support systems in stroke recovery could clarify mechanisms affecting long-term outcomes and help refine rehabilitation strategies.

### Strengths and limitations

This study was exploratory in nature. The lack of differences found within the quantitative results can be understood as identifying areas of commonality in profile and experience. However, it is also possible that with a larger sample size and greater power, some differences would have been found within the quantitative data.

The qualitative part can be criticized because eight participants is a small sample to be able to demonstrate saturation. But even with this limited number, clear gender differences emerged, although more themes might have emerged with a larger number of study participants.

Because of the COVID-19 pandemic, all interviews were conducted by telephone. Telephone interviews can have disadvantages, for example that non-verbal signals, such as facial expression and body language, could not be observed (40). However, earlier studies have shown that telephone interviews are a valid method for data collection (41) and several of our informants also expressed a relief having telephone interview due to easy access.

Another limitation is that self-reported data on QoL and social support may be subject to social desirability bias. Social desirability bias may lead to individuals to overreport positive aspects (such as satisfaction or gratitude) and underreport negative aspects (such as loneliness or distress), especially on sensitive or personal topics (42).

## Conclusion

This study highlights gendered differences in stroke recovery during midlife, underscoring how societal roles and expectations could shape experiences of QoL, coping strategies, and access to support. There was a trend for women to have more persistent problems after their stroke. Despite this, it appeared that men had more difficulty finding their place in their new life situation, while women appeared to demonstrate better coping skills. Men talked about struggles with identity loss, exclusion from social contexts, and greater reliance on work for their QoL, while women discussed a broader range of coping mechanisms and challenges related to balancing responsibilities. The findings suggest that societal and personal expectations regarding gender and roles may intersect with the impact of stroke, influencing recovery pathways. Further research with larger, more diverse samples and different (mixed-method) approaches is needed to validate these findings and explore targeted support mechanisms to improve long-term outcomes for both men and women after stroke.

## Data Availability

The raw data supporting the conclusions of this article will be made available by the authors, on reasonable request. Requests to access the datasets should be directed to marie.materne@oru.se.

## Appendix 1

Interview guide

*Quality of life*:

What is quality of life for you?

How do you experience your quality of life today compared to before the injury?

What are your hobbies?

What were your hobbies before the injury?

Do you have any support to be able to pursue hobbies, in which case, what support?

*Participation and treatment*:

How do you experience your participation today compared to before the injury? (Association life, relationships, leisure, social contexts).

What support do you have today to increase your participation?

How do you feel that accessibility works for you to be able to participate? (Physical availability, information availability, communication availability).

Do you use any aids in your everyday life? Which? (Walker, wheelchair, work chair for the kitchen other).

Do you use your aids?

How do you feel that people around you treat you today compared to before the injury?

How do you feel it works with social relationships today compared to before the injury? (From friends, from your close relatives?).

*Resilience:*

How important have the following factors been when you think about your “recovery journey” after the stroke?

- That you have the support you have needed from others.
- That you have found strength or meaning in life from spirituality.
- That you have an ability not to give up when things get difficult but to keep trying to achieve the goals you have set.
- That you can solve the problems you face.
- That you have hope for the future.
